# Ribozyme-mediated gene knock down strategy to dissect the consequences of PDGF stimulation in vascular smooth muscle cells

**DOI:** 10.1186/1756-0500-5-268

**Published:** 2012-07-10

**Authors:** Caterina Lande, Claudia Boccardi, Lorenzo Citti, Alberto Mercatanti, Milena Rizzo, Silvia Rocchiccioli, Lorena Tedeschi, Maria Giovanna Trivella, Antonella Cecchettini

**Affiliations:** 1 Institute of Clinical Physiology, CNR, Pisa, Italy; 2 Department of Human Morphology and Applied Biology, University of Pisa, Pisa, Italy; 3 Center for Nanotechnology Innovation @NEST, Istituto Italiano di Tecnologia, Pisa, Italy; 4 Istitute of Clinical Physiology, National Research Council – IFC-CNR, Via G. Moruzzi, 1, Pisa, 56124, Italy

**Keywords:** Hammerhead ribozyme, Vascular smooth muscle cells, Cardiovascular disease, Functional proteomics

## Abstract

**Background:**

Vascular Smooth Muscle Cells (VSMCs), due to their plasticity and ability to shift from a physiological contractile-quiescent phenotype to a pathological proliferating-activated status, play a central role in the onset and progression of atherosclerosis and cardiovascular diseases. PDGF-BB, among a series of cytokines and growth factors, has been identified as the critical factor in this phenotypic switch. In order to obtain new insights on the molecular effects triggered by PDGF-BB, a hammerhead ribozyme targeting the membrane receptor PDGFR-β was applied to inhibit PDGF pathway in porcine VSMCs.

**Findings:**

Ribozymes, loaded on a cationic polymer-based vehicle, were delivered into cultured VSMCs. A significant impairment of the activation mechanisms triggered by PDGF-BB was demonstrated since cell migration decreased after treatments. In order to functionally validate the effects of PDGFR-β partial knock down we focused on the phosphorylation status of two proteins, protein disulfide isomerase-A3 (PDI-A3) and heat shock protein-60 (HSP-60), previously identified as indicative of VSMC phenotypic switch after PDGF-BB stimulation. Interestingly, while PDI-A3 phosphorylation was counteracted by the ribozyme administration indicating that PDI-A3 is a factor downstream the receptor signalling cascade, the HSP-60 phosphorylation status was greatly increased by the ribozyme administration.

**Conclusion:**

These contradictory observations suggested that PDGF-BB might trigger different parallel pathways that could be modulated by alternative isoforms of the receptors for the growth factor. In conclusion the knock down strategy here described enables to discriminate between two tightly intermingled pathways. Moreover it opens new attractive perspectives in functional investigations where combined gene knock down and proteomic technologies would allow the identification of key factors and pathways involved in VSMC-linked pathological disorders.

## Background

Coronary artery disease (CAD) is a pathological status induced by the reduction or block of the bloody flow caused by atherosclerotic plaques narrowing or obstructing the arterial lumen. It is well-known that VSMCs play a central role in the onset and progression of the disease and also in the complications observed after Percutaneous Transluminal Coronary Angioplasty (PTCA) that represents the most frequent clinical approach to CAD. Indeed, VSMCs may change their quiescent-contractile physiological phenotype, once subjected to external stimuli, such as cytokines and growth factors, and acquire an activated state endowed with proliferative and migratory properties.

Among the activating growth factors, PDGF-BB, produced by activated platelets and macrophages, has so far been the only factor demonstrated to selectively and directly promote the VSMC phenotype switch [1,2]. The role of PDGF receptors has been described in post injury models [3] and it was detected in human coronary arteries following balloon angioplasty [4]. As a consequence of their induced activation, VSMCs migrate to the sub-intimal space, proliferate, and secrete abundant amounts of extracellular matrix, which forms the bulk of the intimal hyperplastic lesion contributing to restenosis [5].

A series of conventional cytostatic drugs has been adopted to prevent VSMC proliferation and migration after PTCA but a consistent percentage of side effects has been described [6]. More selective nucleic acid-based drugs have been proposed and experimented [7]. Among these drugs, the most frequently exploited ones are ribozymes and siRNAs. Both these inhibitors are interesting since they may be tailored to target and inhibit selectively the genes of interest. The last decade has been characterized by an extraordinary siRNA uptrend, due to the low cost and easy availability. Nevertheless, siRNAs display some disadvantages, since they may saturate cellular machineries and induce off-target effects [8]. Conversely, ribozymes, although more complicated and time consuming in the design, synthesis and validation, are self-acting and more specific.

Hammerhead trans-acting ribozymes are the smallest RNA molecules capable of endonucleolytic activity that specifically bind and cleave RNA sequences of definite targets. Impressively, these precise molecular tools are able to discriminate between targets differing by a single nucleotide [9] and this is the main reason why they have been repeatedly proposed as therapeutic agents also in the field of cardiovascular diseases [10]. In the last years, a different application of ribozymes has stood out with the impressive development of proteomics technologies.

This paper describes the design and experimentation of a hammerhead ribozyme-mediated gene knock down strategy able to dissect PDGF-BB signaling pathway in order to evidence how a single receptor can activate alternative signaling cascades thus emerging as important switch in VSMC phenotype modulation.

## Findings

### Methods

#### Oligonucleotides sequences, synthesis and purification

Selection of target sequence within the porcine PDGFR-β [sequence accession number AF347050S1] and of related minimal ribozyme has been performed according to our previously described predictive method [11]. Selected sequences were: active hammerhead ribozyme, 5′ UCC UUG CUG AUG AGG CCG AAA GGC CGA AAC CAU AU 3′, inactive hammerhead ribozyme, 5′ UCC UUG CU**A** AUG AGG CCG AAA GGC CGA AAC CAU AU 3′ (in bold the inactivating mutation) and short mRNA substrate, 5′ GUC AUA UGG UUC AAG GAC AAC 3′, containing the cleavage target triplet GUU mimicking the position 288–290 of the mRNA. Syntheses have been performed by phosphoramidite chemistry in an AB 3400 synthesizer (Applied Biosystems) using a home-adapted coupling protocol. Crude oligonucleotides were de-protected by treatment with 2 mL of a 1:3 (v/v) mixture of ethanol and 33% ammonium hydroxide (Sigma Aldrich) at 55°C for 12 h and then concentrated to dryness. Crude products were desalted by gel-filtration chromatography in a Sephadex G25 column run in sterile water. Oligonucleotides were then purified by semi-preparative anion-exchange HPLC chromatography on 1.6x10 cm Source® 15Q column (GE Healthcare) eluted at 6 mL/min with 30 min linear gradient from 0.25 to 0.75 M NaCl in 20 mM Tris–HCl buffer pH 9.0 supplemented with 10% acetonitrile (v/v). A recovered fraction, was desalted, as before, by Sephadex G25 gel-filtration chromatography. The freeze-dried collected products were treated with 1:3 (v/v) solution of N,N-dimethylformamide and triethylamine trihydrofluoride (Sigma Aldrich) to remove the 2′O tetrabutyldimethylsilylic protective group. Free products, desalted once again by gel-filtration, were UV quantified at 260 nm absorption measurements in a UV–VIS spectrophotometer (FluoSTAR) before use. A dye-labeled 25mer DNA oligonucleotide (*OligoF*) was also synthesized.

#### In vitro measurement of kinetic constants

A multiple turnover kinetic assay was performed in standard conditions (37°C, pH 7.4, [Mg^+2^] = 10 mM) using a constant 0.2 μM ribozyme concentration and variable amounts of substrate in order to obtain ratios of ribozyme to substrate of 1:10, 1:40, 1:120, 1:180. Stock Ribozyme substrate solutions were separately prepared in a reaction buffer containing 10 mM MgCl_2_, and then heated at 95°C for 2 minutes to denature any secondary structure of RNA molecules. Cleavage reactions were started by adding a constant amount of ribozyme to the substrate solutions at 37°C. At fixed time point (1′, 2′, 3′, 6′, 9′, 12′, 15′, 20′) aliquots were drawn from the reaction mixture and the reaction stopped by adding 50 mM EDTA to abolish the Mg^+2^ contribute to ribozyme activity. Recovered fractions were then analyzed by ion-exchange liquid chromatography using a 4 × 250 mm DNA-Pak® PA-200 column (DIONEX). Runs were performed in a “200 series” apparatus (Perkin Elmer) at 50°C with a flow rate of 1 mL/min under a 30 min linear gradient from 100 to 340 mM sodium perchlorate (Sigma-Aldrich) in 20 mM Tris–HCl buffer pH 9.5 supplemented with 10% acetonitrile. UV signals (recorded at 260 nm) enabled us to quantify the full-length substrate and cleavage products.

#### Synthesis of the vehicle A41-PEI

A two-step synthesis was performed according to our previous experience [12]. Firstly, 6.5 nmol of Polyethylenimine (PEI) (MW 25 KDa Sigma-Aldrich) in PBS buffer pH 7.2, were reacted at 37°C for 3 h with 650 nmol of the bifunctional succinimidyl 4-[N-maleimidomethyl]cyclohexane-1-carboxylate (SMCC) crosslinker dissolved in DMSO. Unreacted SMCC was removed by 10000 molecular weight cut-off centrifugation at 10000 rpm for 20 min at 18°C (Microcon YM10, Millipore). The maleimide-activated PEI was then reacted overnight at room temperature with 650 nmol of the cystein modified third helix homeodomain fragment of the Drosophila Melanogaster antennapedia transcription factor (A41): **C**RQIKIWFQNRRMKWKK (Espikem, Italy). Final purification was obtained by further 45 min cut-off centrifugation at 10000 rpm , at 18°C, using the same centrifugal filter as before in order to discard any unreacted A41. The purified A41-PEI vehicle was quantified by absorption measurement at 280 nm (CARY 100, Varian).

#### VSMC isolation and cultures

Coronaries were dissected from the myocardium of 8-month-old domestic pigs (*Sus scrofa domestica*) obtained from a local slaughterhouse (operating under the local institutional legislation according to ethical directives). VSMCs from tunica media were isolated by enzymatic digestion according to the method described by Christen et al. [13] and cultured in DMEM HG (GIBCO) medium containing 10% FBS (Fetalclone). All reported experiments were performed using cells between the second and sixth passage.

#### Oligonucleotide transfections into VSMCs

The oligonucleotide delivery to cells was performed using the described A41-PEI vehicle. The oligonucleotide amount to be charged onto vehicle, tested under variable N/P ratio (theoretical amine to phosphate groups ratio), was optimized by using the fluorescent *OligoF*. VSMCs were seeded in 12-well plates at a density of 100,000 cells/well for transfection experiments. After 24 hr, fresh medium, supplemented with 10% FBS and containing oligonucleotide-loaded vehicle, corresponding to 250 nM of ribozyme or *OligoF*, was added to the wells. 24 hr later, the cells were, washed in PBS and then either cultured for biological assays or fixed for immunofluorescence.

#### Immunofluorescence analysis

Immunofluorescence analyses were carried out on cells grown and transfected on coverslips. The cells were fixed at 4°C for 30 min with 2% paraformaldehyde in PBS and the fluorescence images were recorded using a DMLB microscope (Leica Microsystems).

#### Quantitative real-time PCR

Primers designed to produce a 137 bp amplification product from PDGFR-β mRNA encompassing the cleavage site of hammerhead ribozyme were: forward primer 5′ GACGCCGTGCAA 3′ and reverse primer 5′GACAGCGCGATCTC 3′. Similar size product from β-actin housekeeping reference transcript was designed and relative primers were: forward 5′CCAACCGCGAGAAGATGA 3′ and reverse CCAGAGGCGTACAGGGATAG 3′. Crude RNA was extracted from control (untreated) or 24 hr ribozyme-treated VSMCs using RNAeasy extraction kit (Qiagen). Aliquots containing 1 μg of total RNA were retrotranscribed using QuantiTect reverse transcription kit (Qiagen) according to manufacturer’s instructions. Quantitative real-Time PCR (qRT-PCR) was carried out with LightCycler 480 (Roche Applied Science). Reactions were performed using LightCycler 480 SYBR Green I Master kit (Roche) under standard conditions. Relative quantification of mRNA was calculated with standard curve method. Transcript values were normalized with those obtained for β-actin (internal control). Each point represents the mean ± SE of at least three independent experiments.

#### Cytotoxicity assay

For the determination of cell viability, VSMCs were seeded in 96-well culture plates at a density of 1000 cells in 100 μL of complete medium per well. 24 hr after seeding, cells were transfected as described above and at interval of 24 hr after transfection 20 μL of 3-(4.5-dimethylthiazole-2-yl)-2.5-diphenyl tetrazolium bromide (MTT) reagent (CellTiter 96® Cell Proliferation Assay, Promega) were added to each well. Following 2 hr of incubation at 37°C to allow color development, absorbance was recorded using a 96-well plate reader (Fluostar Omega, BMG Labtech). Viability tests were carried out on untreated as well as treated VSMC cultures using three concentrations (115 nM, 220 nM, 330 nM) of both active and inactive ribozymes. All measurements were performed in triplicate.

#### Wound assay

VSMCs were plated and transfected with 250 nM of active or inactive ribozymes in 12-well plates, as described above. 24 hr after transfection, a straight scratch, simulating a wound, was made on the cell layer using a pipette tip. Thereafter, 10 ng PDGF-BB were added to the wells for every ml of culture medium. 24 hr later, the cells were fixed with formalin and stained with crystal violet. A dedicated image analysis program (Optiquant, Packard Instruments) allowed the quantification of cells that had migrated into the scratch.

#### Chemotaxis assay

Chemotaxis was measured using the Cultrex® Cell Migration Assay (Trevigen Inc.) according to the manufacturer’s instructions. Briefly, this assay utilizes a simplified boyden chamber design with an 8 micron polyethylene terephthalate (PET) membrane. PDGF-BB (10 ng/mL) was used as chemoattractant. Detection of cell migration was quantified using Calcein AM (acetomethylester). Calcein AM is internalized by the cells and intracellular esterases cleave the AM. Free Calcein fluoresces brightly and the fluorescence is used to quantitate the number of cells that have migrated using a standard curve. 50 000 cells were seeded in each well. Plate was read with a Microplate Luminometer (Glomax Multi Detection System – Promega) using a 485 nm excitation and 520 nm emission conditions.

#### 2D-PAGE, mass spectrometry and western blot

Crude proteins were extracted as described by Rocchiccioli et al [14] from VSMCs, treated or not with oligonucleotide inhibitors, stimulated for 10 min with PDGF-BB. Protein content was quantified by standard bicinchoninic acid (BCA) assay. Proteins were separated by isoelectrofocusing (IEF) according to the method originally described in Görg et al. [15] using pre-manufactured strips with an immobilized non-linear pH gradient ranging from 3 to 10 (Amersham Biosciences, Uppsala, Sweden) on a Ettan IPGphor system (Amersham Biosciences). Second dimension was run on a 9-16% sodium dodecyl sulfate - polyacrylamide gel gradient (SDS- PAGE). Gels were stained in ammoniacal silver nitrate according to the method described in Hochstrasser et al. [16]. The analysis of digital images was carried out with Image Master computer software (GeneBio, Geneva, Switzerland) and only those spots with over 3-fold changes in volume after normalization between the different samples were defined as altered.

Electrophoretic spots were excised and proteins were in-gel digested by trypsin. Peptides were analysed by mass spectrometry (4800 MALDI TOF/TOF - Applied Biosystems).Identification of the proteins was carried out using MASCOT search engine version 2.1 (Matrix Science, Boston, MA).

For immunodetection of Tyrosine-phosphoproteins, after separation by 2D-PAGE, proteins were transferred onto nitrocellulose membranes (Hybond-C, Amersham Life Science). Membranes were incubated overnight at 4°C with a mouse anti-phosphotyrosine monoclonal antibody (IgG2b; Santa Cruz Biotechnology) diluted 1:1,000 in 3% BSA, 0.1% Tween-20 in PBS and then with the secondary antibody, HRP-conjugated anti-mouse IgG (Santa Cruz Biotechnology), diluted 1:3,000, for 1 h at room temperature. Chemiluminescence was detected by an ECL^™^ Detection Kit (Amersham Biosciences) following the manufacturer’s protocol.

#### Statistical analysis

Data were statistically elaborated using the unpaired t-test (* P < 0.05, ** P < 0.01, ***P < 0.001). Each result refers to three independent experiments.

### Results and discussion

#### Kinetic characterization and transfection of hammerhead ribozymes

**Figure 1:**
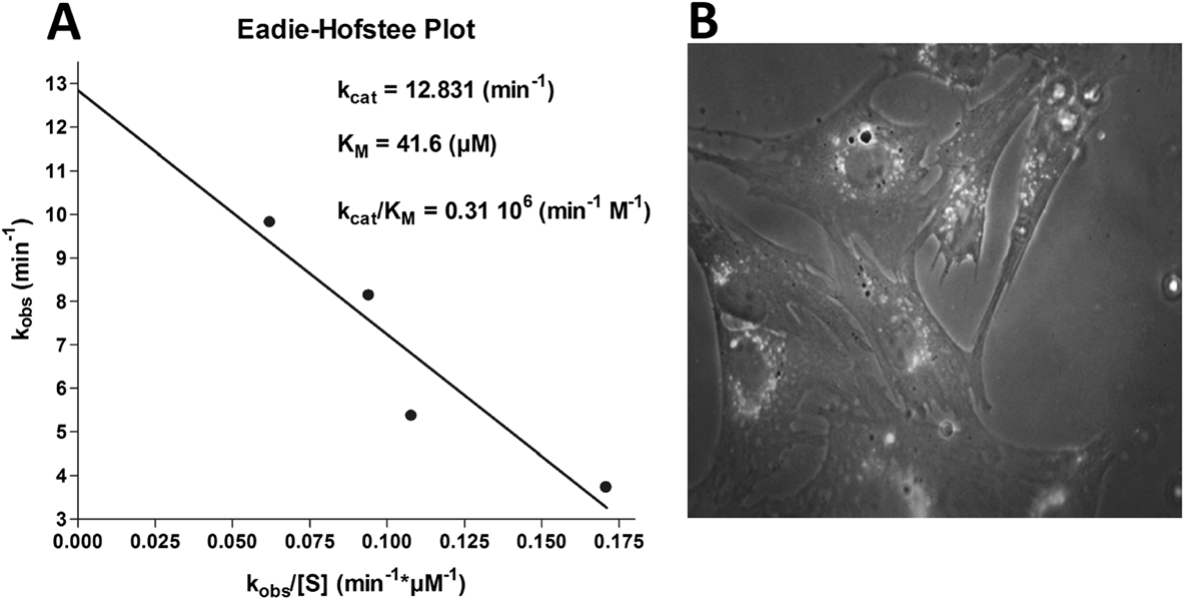
**Kinetic characterization and ribozyme transfection. A**) Graph describing the Eadie-Hofstee interpolation of experimental k_obs_ values (v_0_/[Riboz]) calculated for the in vitro cleavage of synthetic substrate under multiple turnover conditions. The resulting kinetic parameters are also indicated. **B**) fluorescence microscopy image of VSMCs after *OligoF* administration. The intracellular fluorescence demonstrates the oligonucleotide internalization

Although some difficulties due to the fact that gene banks provide only two fragments of the sequence of porcine PDGFR-β messenger RNA (access numbers AF347050S1, AF347050S2), we were able to draw a map of potentially suitable NUH canonical cleavage sites. A GUU triplet starting at nucleotide 288 of the AF347050S1 sequence was selected on the basis of its accessibility indexes (evaluated to be 66.3% for the left binding element and 78.9% for the right element) and of the structural features of the corresponding ribozyme (displaying a calculated −11.3 kcal/mol ΔG° formation free energy value). Both parameters are suitable to provide potentially active ribozymes [17]. Specifically optimized chemical synthesis protocols ensured a > 98% of mean coupling step efficiency and produced a considerable amount (85 nmole; 0.9 mg) of purified product. Ribozyme catalytic activity was monitored by “in vitro” measurements of cleavage kinetics performed on the short synthetic target substrate by an HPLC-based method [18].The direct correlation between the peak area corresponding to target and those corresponding to the products allowed the calculation of the reaction rates and of the kinetic parameters (Figure 1A).

The k_cat_ value of 12.8 min^-1^ is indicative of a high rate of the cleavage step, usually ranging from 1 to 10 min^-1^ for synthetic minimal hammerhead ribozymes. The catalytic efficiency, i.e. the turnover number k_cat_/K_M_, was 0.31 10^6^ min^-1^ M^-1^, a value comparable with those described for other biologically active ribozymes [18,19].

A polymer-based vehicle was designed according to our previous experience [12]. Polyethyleneimine (PEI 25 k) instead of poly-L-lysine was adopted due to its lower toxicity and higher activity as transfecting agent [20]. Moreover, PEI is a commercially available, universally accepted vehicle and can be considered the golden standard among the non-viral transfecting agents. As cell penetrating peptide (CPP) we adopted the third helix homeodomain fragment (A41) of the Drosophila Melanogaster antennapedia transcription factor since it is a characterized by a high efficiency to cross the cell membranes [21,22]. The number of A41 molecules bound to primary amino-groups of PEI ranged around a ratio of 1 to 10 which was previously observed to represent an optimized compromise between delivery effectiveness and toxicity. The ultrafiltration-coupled preparation method of vehicle enabled to obtain a 60% yield from starting reagents.

Transfection efficiency of vehicle was monitored under different N/P ratios (amine group of PEI over phosphate groups of oligonucleotides) exploiting *OligoF* and evaluating the content of incorporated fluorescence into cultured cells by cytofluorimetry. Even under lower N/P conditions (N/P = 6), 48% of cells became fluorescent due to the intake of *OligoF*. A saturation of delivery activity (over the 90% of cells) was reached for N/P higher than 17. Such results demonstrate the active synergistic effect of membrane-fusogenic A41 peptide which improves the ability of the complex “vehicle/oligonucleotide” to cross the cell membrane.

The uptake of the oligonucleotide into the cells was also checked at morphological level by fluorescence light microscopy analyses. Perinuclear fluorescence was evident in 90% of the cells and appeared to be clustered into small roundish vesicles (Figure 1B).

#### PDGFR-β knock down assessment and toxicity of the treatments

**Figure 2:**
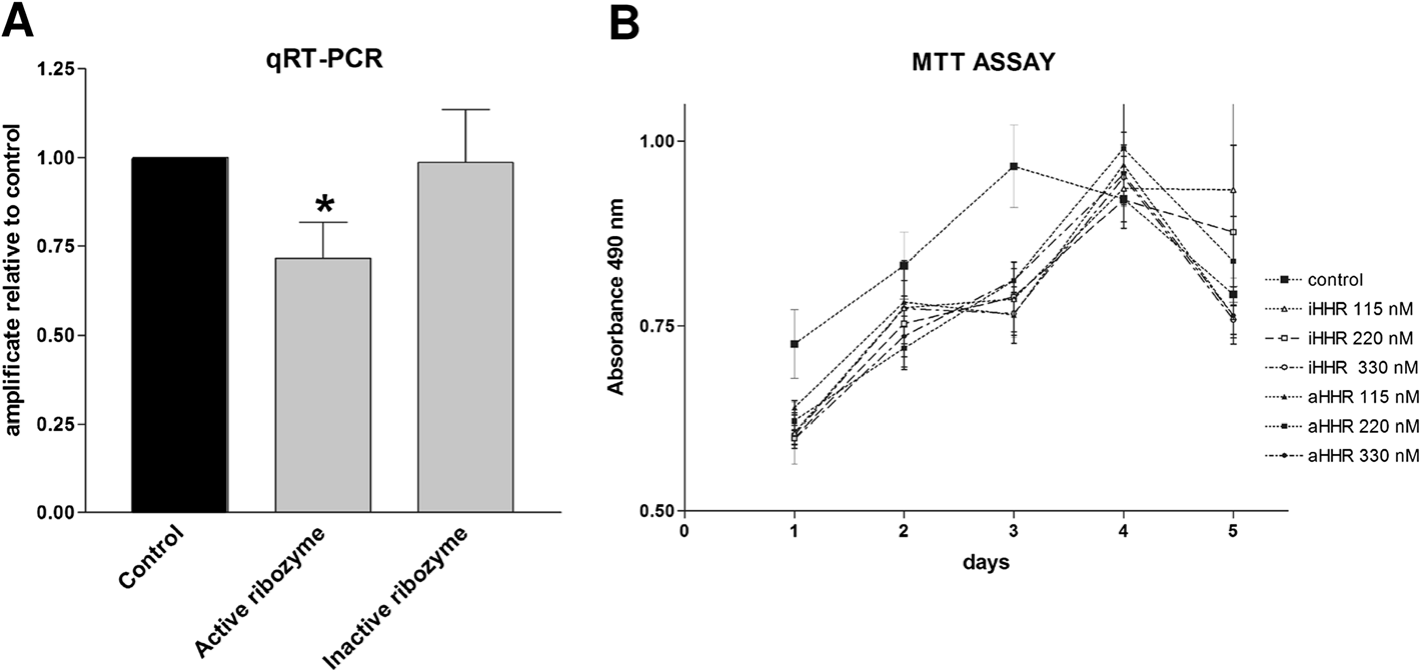
**PDGFR-β knock-down assessment and toxicity. A**) Histogram of Real-time PCR amplification of RNA recovered from VSMCs. Control bar represents the results obtained with VSMCs cultured in complete medium and normalized to 1 as reference value. Gray bars represent mean ± SE of three independent experiments. Statistical significance was calculated applying the unpaired t-test (* P < 0.05). **B**) Graph of summarizing MTT assay. VSMCs were cultured in standard condition for the control or in the presence of active (aHHR) and inactive ribozymes (iHHR). Each point represents the mean ± SD of 3 independent replicates

Relative expression levels of PDGFR-β mRNA as obtained by qRT-PCR have been normalized to reference value 1 for proliferating VSMCs cultured in standard conditions (Figure 2A). Active ribozyme treated cultures display a 30% reduction of PDGFR-β messenger RNA if compared to untreated cells. By contrast, the inactive ribozyme was totally ineffective. Such results demonstrate that the active ribozyme is effective and moreover its inhibitory activity is still present after 24 hr treatment nevertheless the documented vulnerability of short naked RNA. Unlikely, it was not possible to assess the decrease in protein expression due to the unavailability of specific antibodies raised against the porcine antigenes.

Viability tests after treatments with three different amount of active or inactive ribozymes have been performed up to 5 days. Cells were cultured in the presence of the ribozyme bound to the A41-PEI vehicle at constant N/P ratio of 17. Records of the formazan product by absorbance at 490 nm was measured at 24 h intervals (Figure 2B). No substantial differences can be observed either between active and inactive ribozymes also among the different doses used. The slight delay in the curves, observed in all oligonucleotide-treated cultures as compared to controls, might reflect the overall cell perturbation provided by the polyplexed complex (vehicle + oligonucleotide).

#### Effects of PDGFR-β gene knock down on VSMC migration

**Figure 3:**
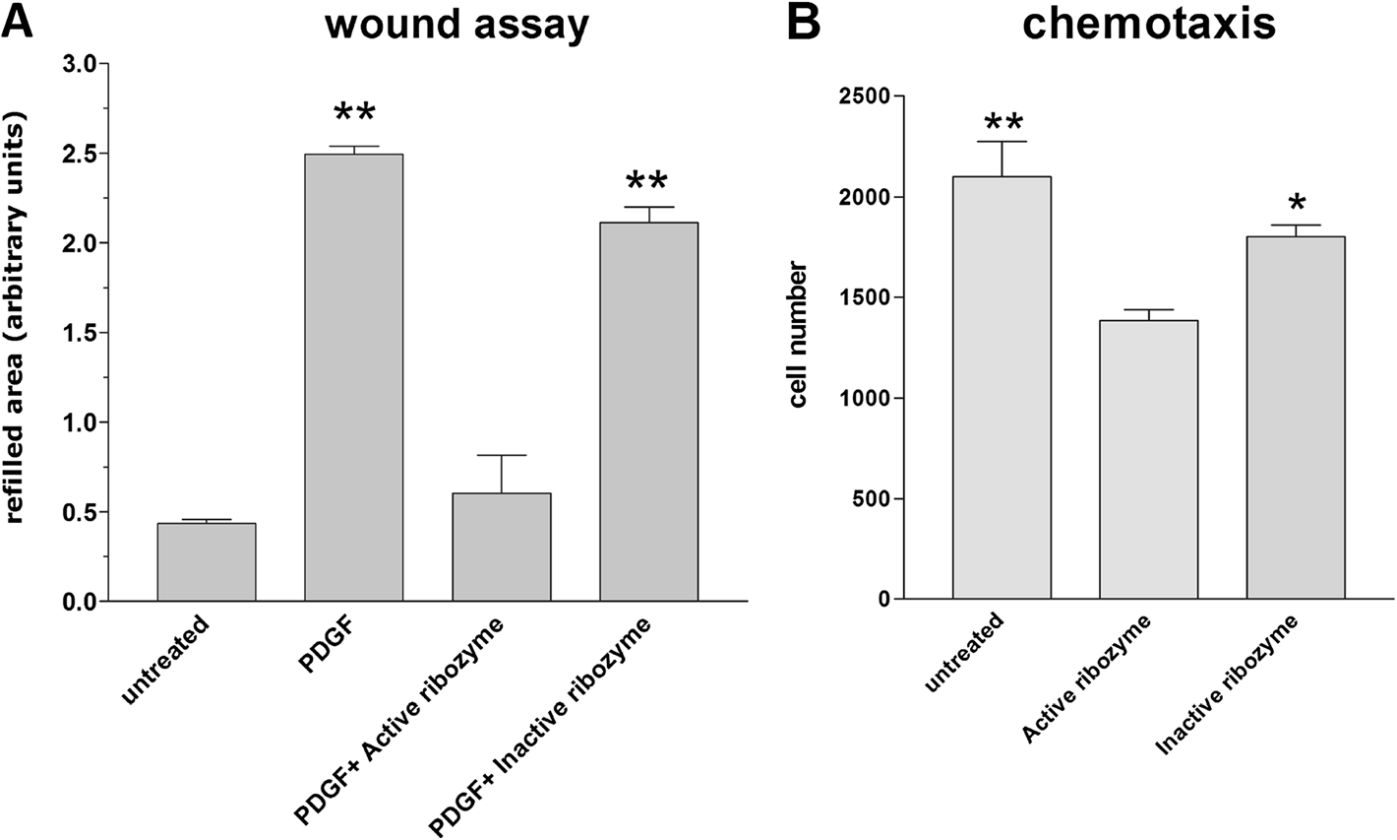
**Migration activity of VSMCs cultured under different conditions. A**)- Wound assay istogram: untreated represents quiescent VSMCs cultured in absence of serum; PDGF accounts for VSMCs stimulated for migration with serum supplemented with PDGF-BB; PDGF+Active ribozyme or +Inactive ribozyme are VSMCs stimulated as before and treated with active or inactive ribozymes. **B**)- Chemotactic activity induced by PDGF-BB on VSMCs. Bars represent the mean ± SE of at least three independent experiments. Data computing the migration activities of VSMCs were statistically elaborated using the unpaired *t-*test (* P < 0.05, ** P < 0.01)

Histogram in Figure 3A shows that stimulation of the cells, obtained by adding PDGF-BB to complete medium, increased six times the effects of migratory activity if compared to quiescent (i.e. cultured in the absence of serum) VSMCs. The administration of active ribozyme resulted in a 90% inhibition of PDGF-BB stimulated repair of wound. On the contrary, the inactive ribozyme produced a considerably reduced effect, namely a 17% inhibition. This last inhibitory activity may depend on the antisense effect of inactive ribozyme as it has been previously suggested [23].

Chemotaxis (Figure 3B) was also analysed in Boyden chambers using PDGF-BB as chemoactrator. The number of migrating cells was significantly reduced of about 32% by the ribozyme administration as compared to untreated or inactive ribozyme treated cultures.

#### Knock down-induced changes on protein phosphorylation

This study was triggered by a previous published work [24] in which PDGF-BB resulted to heavily affect the phosphoproteome profile of VSMCs. Among other factors, heat-shock proteins and other chaperones have been indicated to participate actively to cell remodeling after growth factor stimulation and to contribute to the so called “inside-out” cross talk, responsible of the cell activation [5]. In particular, HSP-60 and PDI-A3 seemed interesting for the assessment of our partial knock down system since HSP-60 has been well demonstrated as being involved in cardiovascular diseases [25,26] with a suggested role in VSMC migration [26,27]. Accumulating evidence has revealed the presence of chaperones (HSP-60 and PDI-A3) on cell surface and it has also proposed that their tyrosine phosphorylation might constitute a possible mechanism for reorientation of receptor complexes and key molecules in the process of cell activation [28-30]. This mechanism is particular interesting in the VSMC phenotypic switch that characterizes their activation and entails important changes in membrane morphology and composition. Major outcome of this phenomenon is the acquisition of a migratory capability. In this respect, it has been reported that PDI-A3 functions at the cell surface, where it may be involved in disulfide exchange required for cell mediated adhesion by integrins [31]. Interestingly, it has also been shown that PDI-A3 can mediate conformational changes in both β1 and β3 integrins, which may lead to cell adhesion to a particular substrate [32]. Moreover, PDI-A3 might be committed with PDGF targeting to its receptors [33]. For all this reasons, PDI-A3 and HSP-60 chaperones were selected in order to validate our knock-down results.

**Figure 4:**
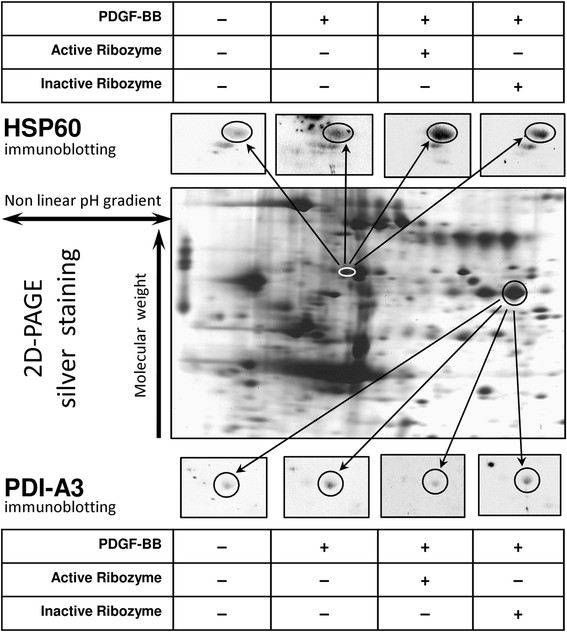
**Proteomic approach to phosphorylation study of PDI-A3 and Hsp-60 chaperones.** Middle panel shows the 2D-PAGE mapping of both proteins after silver staining. Inserts illustrate the immunoblotting results of membranes probed with anti-phosphotyrosine antibody. Upper inserts refer to HSP-60 while lower inserts describe the PDI-A3 staining

After ribozyme treatment and PDGF-BB stimulus, VSMCs were collected, the protein content was extracted and separated by 2D-PAGE. The spots corresponding to PDI-A3 and HSP-60 were identified by mass spectrometry analysis. Following electro-transfer on nitrocellulose membranes, tyrosine-phosphoproteins were immunodetected by a specific monoclonal antibody. Representative images of gel and blots are reported in Figure 4.

**Figure 5:**
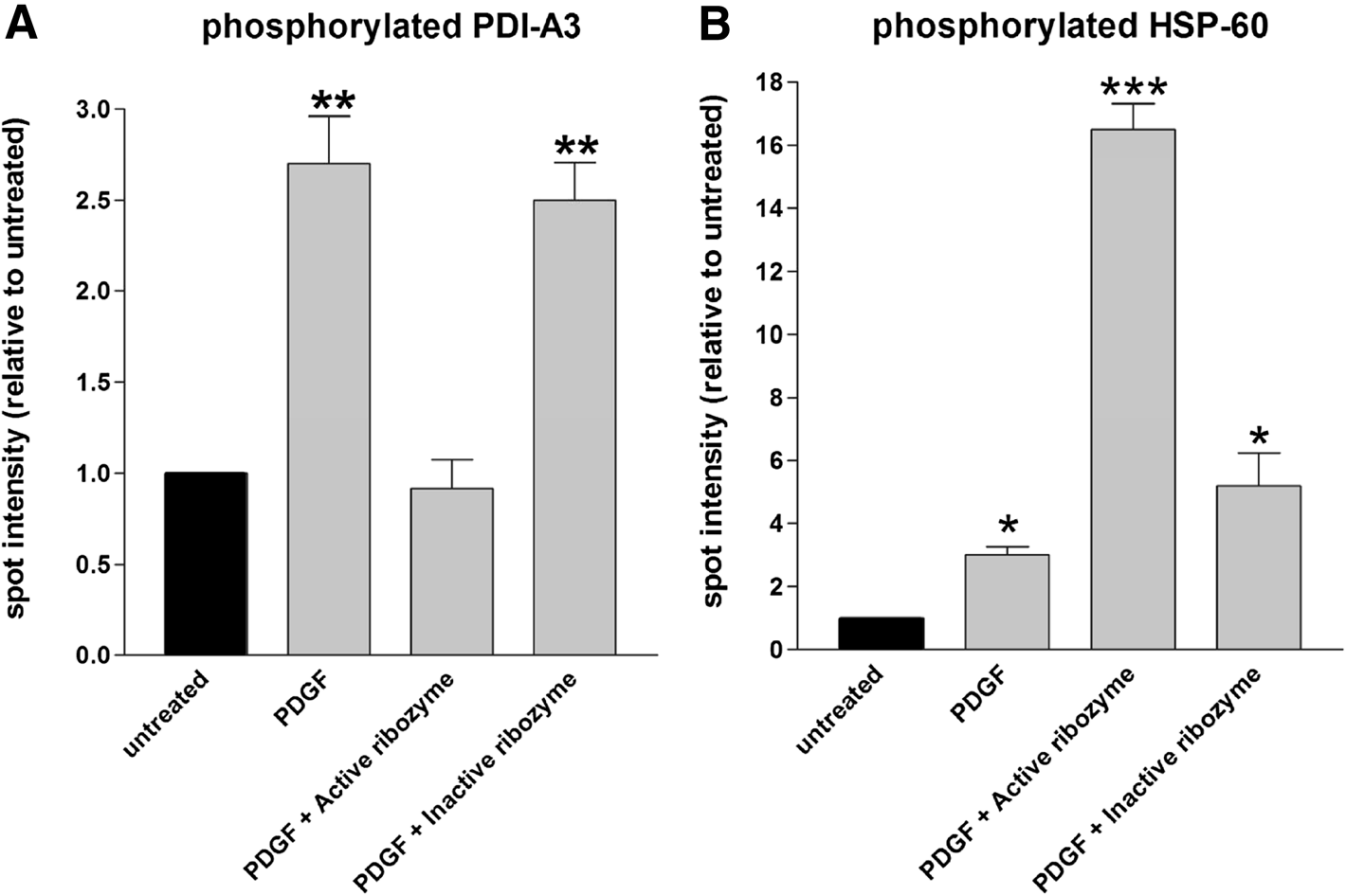
**Phosphorylated protein comparative analyses. A**) Histogram of PDI-A3 phosphorylation . **B**) Histogram of HSP-60 phosphorylation. Untreated: refers to quiescent VSMCs. Gray scale value of untreated samples was conventionally assumed to be 1 as normalization factor; PDGF: refers to VSMCs stimulated with PDGF-BB; PDGF+active ribozyme and PDGF+inactive ribozyme: refer to VSMCs cultured as before and additionally treated with active or inactive ribozyme. Bars represent the mean ± SE of at least three independent experiments. Data were statistically elaborated using the unpaired *t-*test (* P < 0.05, ** P < 0.01, *** P < 0.001)

Histograms in Figure 5, panel A and B, summarize the results. The comparison of identified spots was obtained after image de-noising and normalization to the control samples to which the arbitrary value of 1 was assigned. PDI-A3 phosphorylation increased 2,7 fold after PDGF-BB activation, while the receptor partial knock down abolished this induction. Such effect is highly specific since treatment with inactive ribozyme showed a phosphorylation profile quite close (0.9 fold) to that induced by the growth factor (Figure 5A). Conversely, HSP-60 phosphorylation, is dramatically increased, under partial knock down condition, more than 5 times over the PDGF-BB activated sample and 16 fold over the untreated cells (Figure 5B) while the inactive ribozyme administration produces a reduced phosphorylation level. It is interesting to observe that there is an increase of HSP-60 phosphorylation after the addition of the inactive ribozyme in comparison to the stimulation with PDGF. We believe that this outcome is caused by an already described [23] antisense effect due to the fact that the inactive ribozyme binds its target but it is not able to cut the molecules.

### Conclusion

With the administration of hammerhead ribozymes, a significant reduction of the PDGFR-β target mRNA was obtained. This treatment appeared devoid of any toxic side effects and considerably affected the PDGF-BB signaling activity as revealed by biological assays. Indeed, migration activity in ribozyme-treated cultures, monitored by wound assay, fell down to 10% of levels attributed to PDGF stimulated cells. The specificity of such a knocking down effect was confirmed by the very small inhibition induced by similar administration of the inactive ribozyme.

**Figure 6:**
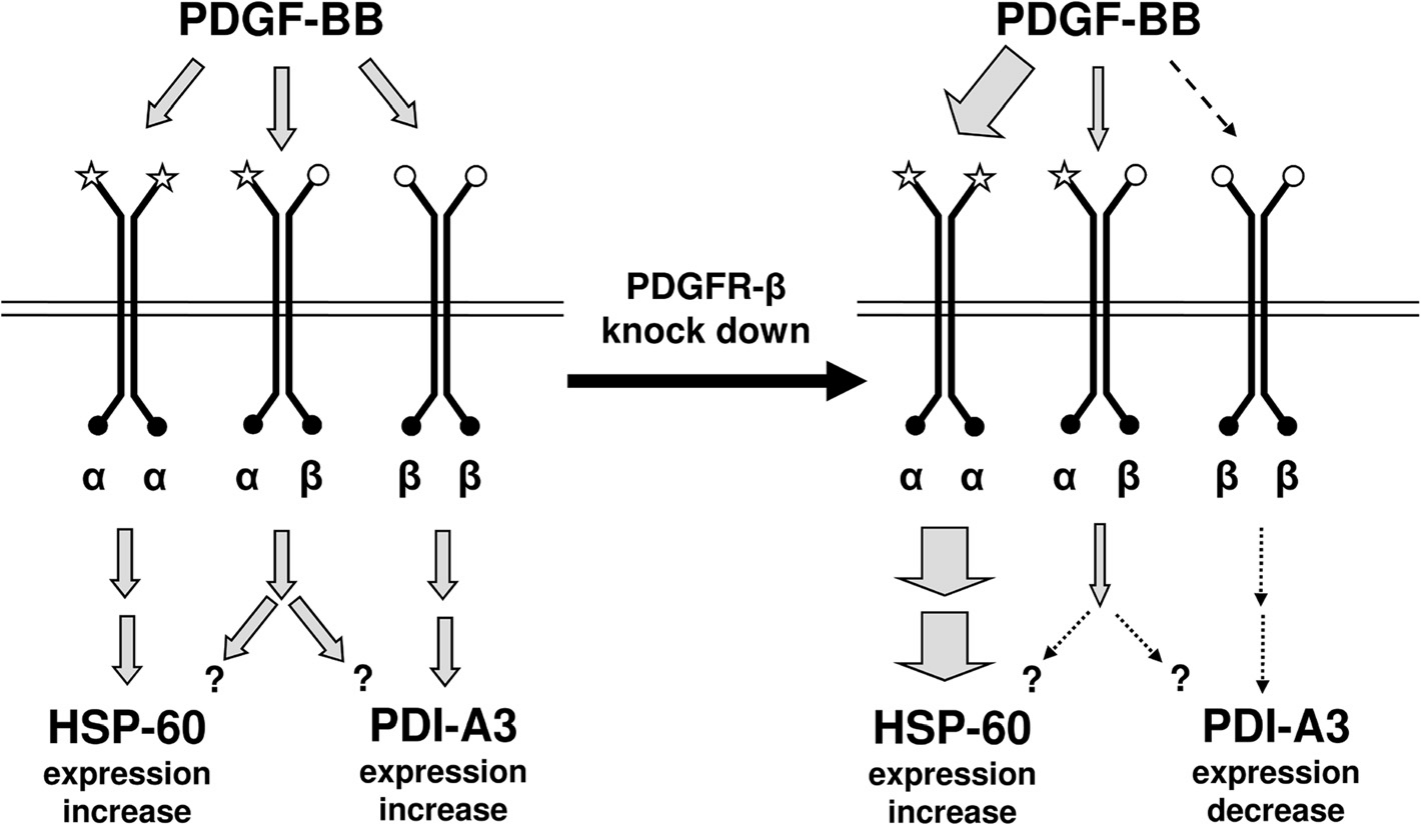
**Working model describing the different effects on PDI-A3 and HSP-60 phosphorylation due to PDGFR-β knock-down.** Ribozyme might impair the β-β and α-β dimer formations decreasing the opportunities of PDGF-BB binding. This inhibition might reduce (if not abolish) the PDGFR-β signaling pathway and conversely increase the PDGFR-α pathway

Interestingly PDGFR-β gene partial knock down produced opposite effects on tyrosine phosphorylation of the chaperones PDI-A3 and HSP-60 although a comparable inductive effect triggered by PDGF-BB on both proteins. A possible interpretation of these data is that the PDGFR-β partial knock down causes an unbalance among the different PDGF receptors. It is well-known that PDGF-BB binds both structurally similar protein-tyrosine kinase receptor subunits (PDGFR-α and PDGFR-β) and that dimerization and autophosphorylation of PDGFR occur upon receptor-ligand interaction. Moreover, differential binding of initial signaling molecules to phosphorylated PDGFRs is thought to mediate overlapping but distinct α- and β-PDGFRs-induced signaling pathways. In murine fibroblasts, it has been demonstrated that PDGF-BB activation of β-PDGFR induces both pro- and anti-transformation pathways, while activation of PDGFR-α promotes transformation pathway through phosphorylation/activation of different signaling molecules [34]. Given that in our experiments the β isoform was specifically knocked down, after treatment, the α-α homodimer would become the prevalent competitor for growth factor binding. Consequently, it is conceivable that PDI-A3 phosphorylation after VSMC activation could depend on signaling triggered by the β-containing receptors (sensitivity to knock down) while HSP-60 phosphorylation could depend on α-containing receptors whose signaling would be prevalent after β isoform removal (Figure 6).

The model described in this figure, once confirmed, would open new attractive perspectives in studies of functional proteomics. Combined proteomic and gene knock down technologies would enable the identification of key factors and key pathways involved in VSMC activation and eventually suggest crucial elements suitable for diagnostic evaluations and useful for planning new therapeutic strategies.

## Competing interests

The authors declare that they have no competing interests.

## Authors’ contributions

CL carried out the design, the synthesis and the purification and kinetic analysis of ribozyme, performed the data interpretation, and the manuscript drafting and revising. CB performed the Western Blotting of phosphorylated proteins. LC was involved in the interpretation of analytical data and in the conceiving of the study. AM was involved in the design of the ribozyme. MR performed the quantitative real-time experiments. SR performed the HPLC analysis. LT was involved in the design and the synthesis of the vehicle. MGT performed animal surgical interventions and coronary explanting according to bioethical guidelines. AC conceived the study, optimized cell culturing and cell activation conditions, coordinated cell explanting treatments and collaborated in all the biological aspects concerning compilation of the manuscript. All authors read and approved the final manuscript.
